# “Knowledge and practices of HIV infected patients regarding medicine disposal among patients attending public ARV clinics in KwaZulu Natal, South Africa”

**DOI:** 10.1186/s12889-020-09018-4

**Published:** 2020-06-08

**Authors:** Pooja Maharaj, Sooraj Baijnath, Panjasaram Naidoo

**Affiliations:** Discipline of Pharmaceutical Sciences, School of Health Sciences, University of KwaZulu-Natal, Westville Campus, University Road, Westville, Private Bag X54001, Durban, 4000 South Africa

**Keywords:** Environmental health, Pharmaceutical waste, Antiretroviral drugs

## Abstract

**Background:**

Recent analysis of drinking water in South Africa found the presence of ARVs, other pharmaceutical and personal care products. The environmental and human health risk that this presents is daunting. The increased use of ARVs with poor disposal practices could be the reason for these substances being present in drinking water. Therefore, this study aimed to determine the knowledge and practices of HIV infected patients, regarding medicine disposal.

**Method:**

A descriptive, cross sectional, quantitative study that utilised a structured, self-administered, questionnaire was undertaken at 3 different public ARV clinics in the eThekwini Metro of KwaZulu Natal, SA. The variables included questions on demographics, knowledge and practices of medicine disposal of ARV and other medications. Data was captured using excel spreadsheets and analysed using SPSS version 25. Chi square tests were used to compare factors between correct and incorrect knowledge and practice groups.

**Results:**

Four hundred and eighty four participants agreed to participate in this study, of which the majority (71.1%) were females. Over 87% of the participants knew that improper disposal of medicines were harmful to the environment with only 28.3% knowing that there were laws governing the way medicines should be disposed. Majority of participants that had unused and expired ARVs at home disposed of these medicines. The most common route of medicine disposal for ARVs was by throwing these medicines into the bin (56.4%). Only 24.2% of participants were informed by healthcare professionals about the proper method of medicine disposal. Participants who had secondary and tertiary level of education (*p* = 0.043) and the ability to speak English (*p* = 0.001) had appropriate knowledge on medicine disposal.

**Conclusion:**

This study identified that poor medicine disposal practices and lack of adequate information about the proper methods of medicine disposal were evident among the participant population. There is a need for patient education and healthcare professional intervention to ensure patients are aware of standard proper medicine disposal practices.

## Background

South Africa has the greatest number of people on antiretroviral drugs globally, with approximately 7.7 million people living with HIV (Human Immunodeficiency Virus) and KwaZulu Natal having the highest HIV prevalence rate in South Africa [1]. The scale of the treatment programme being so huge could lead to challenges in terms of the environment and human health. Recent analysis of drinking water in South Africa has found that pharmaceuticals and personal care products, such as antibiotics, antidepressants, artificial sweeteners, illegal and antiretroviral (ARV) drugs are becoming increasingly common contaminants in drinking water [2]. These contaminants could lead to detrimental health effects such as contributing to drug resistance, genetic effects, renal failure and impairment of sexual development [3], as well as impacting the growth, fertility and behaviour of other organisms in our ecosystem [4].

The possible ways that ARVs get into the waters could be via human faeces, urine and/or improper disposal of unused and expired medicines [2]. Of concern is that often HIV patients are on more medication than just their ARVs, due to immune-suppression that makes these patients susceptible to opportunistic infections [5], in which case these medicines could be experiencing the same disposal fate as that of the ARVs and entering the water system. HIV infected patients may not adhere to their antiretroviral treatment (ART) due to various reasons, hence, resulting in an accumulation of medicines. If there is no knowledge on proper disposal methods, then these medicines are found harming the environment.

Inappropriate disposal routes such as the sink, toilet or rubbish are important factors that are contributing to water and ground contamination and the environmental occurrence of pharmaceutically active compounds, which are difficult to remove using traditional wastewater treatment processes [6]. This could result in ARVs being consumed in drinking water and the possibility of the development of resistance [7], accidental consumption by children resulting in accidental poisoning [8] and accumulation of these medicines in aquatic biota directly from the water [7]. Studies have shown that the majority of patients discard their medication by throwing it in the trash or by flushing it down the sink or the toilet [3, 9–11]. A study done in the United States, found that 53.8% of patients, presenting at outpatient pharmacies, flushed their unused and expired medicines down the toilet and 35.2% rinsed them down the sink, which meant that among the entire population of participants, 89% of their unused and expired medicines were being disposed of in a manner in which they could enter the water system [9]. Similarly, a study done in Kuwait, also found that among patients at general public hospitals, 76.5% disposed of their unwanted medicines in the trash and 11.2% flushed it down the toilet, a total of 87.7% disposing of medicines in an improper manner [10].

In South Africa the disposal of medicines is prescribed in terms of Regulation 27 of the Medicines and Related Substances Act 101 of 1965 where the requirement is that medicines should be disposed of in a manner that is irretrievable [12] and not to be disposed in municipal sewerage systems. Unlike other countries that use drug take back programmes that run at specific periods during the year, South Africa has policies in place that allow patients to return their unused and expired medicines to any pharmacy at any time. Pharmacies store these medicines and employ third party waste management companies to dispose of these medicines [12, 13], primarily through incineration. The intention is to ensure that there is no harm to the environment or health of humans [14].Therefore, patients should be informed and requested to return any unused or expired medicines to a pharmacy or health care professional for safe disposal.

However studies have shown that often patients are aware on how to use and store the medication but rarely are they informed about the disposal of such medications [15]. A study done in KwaZulu Natal, South Africa found that most people are knowledgeable about effective medicine storage, however, there is a lack of adequate information on safe medicine disposal and this could lead to negative consequences [16]. Similarly a study in Saudi Arabia, in 2017, found that 84% of the outpatient population at a health centre never received any information from their healthcare providers about safe and proper disposal of medication [15]. Most studies concluded that there is a requirement for the establishment of uniform guidelines and education on the safe disposal of unused and expired medicines amongst the public [3, 9–11, 15, 17, 18]. Therefore, it is clear that patients are lacking information about the proper disposal method and educating patients about safe disposal of their medicines is a crucial step to limit the presence of pharmaceutical waste in the environment.

In addition, some patients often have different beliefs about the way to dispose of pharmaceutical waste than the proper method of disposal. One study found that 21 and 35.7% of the patient population found it acceptable to dispose of medicines by rinsing it down the sink and flushing it down the toilet respectively [9]. A study done in Kabul, in 2017, among the general population found that 98% of people felt that improper disposal of unused and expired medicines can affect the environment and people’s health [3]. Hence, there is knowledge and understanding about the effect of improper disposal, however, there seems to be lack of knowledge about the appropriate methods of disposal. This lack of knowledge calls for appropriate healthcare professional intervention, in terms of providing valuable education on how to use and dispose of unwanted pharmaceutical waste in an effective manner [9, 15, 19]. Healthcare professionals who engage with patients should ensure that they provide counselling on the disposal of unwanted medicines and in the case of HIV patients adherence to ARV should be emphasised. There is a dearth of studies done on patients’ knowledge and practice of medicine disposal in South Africa. No study has focused on public sector HIV infected patients’ knowledge and practices of medicine disposal. Due to the lack of data on this important area of environmental health a study was undertaken to identify to what extent unused and expired ARVs, as well as other unused and expired medicines, are disposed of inappropriately, ascertain the knowledge of HIV patients on safe disposal of medicines and identify if healthcare professionals are informing HIV infected patients about the effective methods of medicine disposal.

## Method

### Study design

This study was a descriptive, cross sectional, quantitative study.

### Study setting

Three ARV clinics attached to 3 public sector hospitals in the eThekwini Metro of KZN, South Africa was selected for the study. KwaZulu Natal is a province located in South Africa with an estimated population size of 11065240 [20]. There are 10 districts with eThekwini as the metropolitan. South Africa has a two tier healthcare system that comprises of a public sector (run by the government) and a private sector. The public sector health services are divided into primary, secondary and tertiary healthcare facilities. ARV clinics fall under the primary healthcare facilities, primarily run by nurses.

### Study population and sample size

The study population included all patients 18 years and above or caregivers 18 years above that visited the selected ARV clinics. To estimate knowledge and practices of patients regarding disposal of medicines in public ARV clinics, assuming 95% confidence and an acceptable margin of error of 5%, and maximum variability i.e. 50% (given unknown previous knowledge and practices) a sample size of 385 participants was calculated. The sample was further increased by a margin of 25% to account for potential non-responses, resulting in a minimum sample size of 482 required. Persons who were willing to participate in the study and gave consent were recruited into the study. A non-probability convenience (availability) sampling technique was used to reach the sample size.

### Inclusion criteria/exclusion criteria

All persons 18 years old and above who were either HIV positive or took care of a HIV infected person was included in the study.

All persons younger than 18 years old who were neither HIV positive nor taking care of a HIV infected person was excluded from the study.

### Research instrument

A structured self administered, anonymous, coded questionnaire was developed. It contained three sections viz. Demographics which included questions on the age, gender, home language, socio-economic status of the participants. The second section was on knowledge, where participants knowledge was defined as knowing that the proper method of medicine disposal was the hospital or pharmacy and the final section had questions on participants practices. The questionnaire was developed using current literature [3, 15, 21] and adapted to meet the objectives of this study (face validation). The questionnaire was given to 5 postgraduate (PG) pharmaceutical science students who were familiar with the topic to check for ambiguity of questions and the time it took to complete the questions. The questionnaire was also given to 2 academics from the Discipline of Pharmaceutical Sciences, at the University of KwaZulu Natal, who were experts in questionnaire construction. Inputs from the PG and academics were used to amend the questionnaire. Each section gave the participant options to choose from by simply marking a tick or cross next to their preferred choice. The questionnaire was available in English and IsiZulu as they are the most common languages spoken in the province of KwaZulu Natal.

### Questionnaire administration and data collection

The calculated minimum sample size of 482 was divided amongst the 3 selected ARV clinics giving a total of between 161 and 162 participants per clinic. Data was collected between July 2019 and August 2019.On the day of data collection, the researcher addressed all the patients/caregivers who were awaiting medical attention. This method of recruitment was employed to exclude selection bias. Over a period of 8 days, spent at each clinic, a total of 400 people (per clinic) were approached to participate in the study. When the sample size of 161/162 was obtained, per clinic, recruitment drive had stopped and the researcher moved to the next clinic, where the same process was employed, that is, the study was first explained to the patients/caregivers and they were invited to participate. If they agreed to participate, an introductory letter and an informed consent form was given to them. The introductory letter provided more information on the study and also emphasised that even after agreeing to participate they could still withdraw from the study without suffering any penalties or prejudices and that all information will remain confidential. On receipt of a signed informed consent form, a questionnaire was given to the participant in their preferred language, either in isiZulu or English.

This being a voluntary participation study, the researcher did not request any reasons from the patients/caregivers as to why they did not want to participate in the study. The researcher assisted participants who were illiterate by reading the questions and ticking their responses on the relevant questionnaire box.

### Data capture and data analysis

The data was captured from the completed questionnaires onto excel spreadsheets and imported onto SPSS version 25 for statistical analysis. Descriptive statistics were used to summarize the data while the factors associated with knowledge and practices were tested using chi-square tests or Fisher’s exact tests as appropriate for independent categorical variables at a 0.05 level of significance.

### Ethical considerations

Ethical approval was obtained from the Biomedical Research Ethics Committee (BREC) of the University of KwaZulu-Natal. [BREC Reference: BE 260/19].

## Results

Only valid percentages have been reported in the study.

### Response rate

From a total of about 1200 persons that were approached to participate, 484 persons responded positively to the request, which exceeded the calculated valid sample size of 482. All 484 who responded returned a completed questionnaire.

### Demographic of participants

Of the 484 participants the majority were females (*n* = 344; 71.1%) with the most predominate overall age group being 31 and above (*n* = 365; 75.4%). More than half of the participants (*n* = 298; 62.0%) had secondary schooling (Table 1).

**Table 1:** Demographics of the participants (*n* = 484)

		Number of participants Cumulative % (N)	Valid Percentages
**Gender**	Male	28.7% (139)	28.7%
	Female	71.1% (344)	71.1%
	Transgender	0.2% (1)	0.2%
**Age**	18–20	6.2% (30)	6.2%
	21–30	18.4% (89)	18.4%
	31- above	75.4% (365)	75.4%
**Education**	Missing responses	0.6% (3)	
	no formal schooling	6.6% (32)	6.7%
	Primary	17.1% (83)	17.3%
	Secondary	61.6% (298)	62.0%
	Tertiary	14.0% (68)	14.1%

### Other demographic data of participants

Majority of the participants were able to understand isiZulu (*n* = 447; 92.4%) and English (*n* = 296; 61.2%). A total of 17 languages were indicated as spoken by participants (English, isiZulu, Tsonga, Afrikaans, Tswana, Swati, Southern Sotho, Northern Sotho, Ndebele, Xhosa, Venda, French, Hindi, Shona and Tsingisi).

Nearly, half (42.1%) of the participants lived in an urban area with 58.1% living in a formal house. Over 96% of participants had easy access to water with 81.8% having water inside their house and 68.0% having flush toilets at home.

Participants were taking a median of 2 tablets per a day with a range from 1 to 14 tablets.

### Knowledge about medicine disposal

Participants knowledge was defined as knowing that the proper method of medicine disposal was the hospital or pharmacy. (Table 2) When participants were asked what they thought was the best method to dispose their unused and expired medicines the most common response amongst the participants was throwing medicines into the bin (44.2%). Nearly a third of participants chose the pharmacy (31.8%) and hospital (33.6%) to return their unused and expired medicine. Overall more than 62% (62.8%) of the participants did not know how to properly dispose of their unused and expired medicines.

**Table 2:** Participants response to known medicine disposal routes

	Participants responses	Number of participantsCumulative% (N)	Valid Percentage
Throw medicines into the bin	Missing responses	7.9% (38)	
	Yes	40.7% (197)	44.2%
	No	51.4% (249)	55.8%
Throw medicines into the toilet (PIT)	Missing responses	8.5% (41)	
	Yes	37.8% (183)	41.3%
	No	53.7% (260)	58.7%
Throw medicines into the Sink	Missing responses	8.9% (43)	
	Yes	15.7% (76)	17.2%
	No	75.4% (365)	82.8%
Throw medicines in the Sand	Missing responses	9.7% (47)	
	Yes	14.3% (69)	15.8%
	No	76.0% (368)	84.2%
Throw medicines in the Bush	Missing responses	11.0% (53)	
	Yes	4.5% (22)	5.1%
	No	84.5% (409)	94.9%
Flush the medicines	Missing responses	9.3% (45)	
	Yes	31.4% (152)	34.6%
	No	59.3% (287)	65.4%
Give medicines to friends/family	Missing responses	10.1% (49)	
	Yes	3.5% (17)	3.9%
	No	86.4% (418)	96.1%
Pharmacy	Missing responses	7.6% (37)	
	Yes	29.3% (142)	31.8%
	No	63.0% (305)	68.2%
Hospital	Missing responses	7.6% (37)	
	Yes	31.0% (150)	33.6%
	No	61.4% (297)	66.4%
Other	Missing responses	99.8% (483)	
	Yes	0.2% (1)	100%
	No	0.0% (0)	0%

Four hundred and twenty participants (87.8%) agreed that medicines were harmful to the environment if disposed of inappropriately and only 28.3% of participants were aware that there are laws in South Africa that govern the way medicines should be disposed of.

### Role of healthcare professional

More than half of the participant population (*n* = 363; 75%) stated that they were never informed by their healthcare professional on how to dispose of unused and expired medicines effectively and 0.8% were not sure. Of the 24.2% of participants that were informed, most of the participants (59.8%) were informed by a nurse (Table 3).

**Table 3:** Participants that were informed by healthcare professionals about the proper method of medicine disposal

Healthcare professional	Number of participants % (N)
Nurse	59.8% (70)
Doctor	30.8% (36)
Pharmacist	11.1% (13)
Other	9.4% (11)

### Factors affecting participants knowledge

Chi square tests were performed to identify factors that influenced participants knowledge. Overall 37.2% of participants knew how to dispose of their unused and expired medicines appropriately. Those who spoke English were more likely to have proper knowledge on medicine disposal (*p* = 0.001) and those with secondary or tertiary education were more likely to have knowledge as opposed to the other education levels (*p* = 0.043). Participants who kept their unused medicines were more likely to know the correct method of medicine disposal (*p* = 0.041).

### Participant’s disposal practices

Of the 484 participants on ARV medication, 139 (28.7%) participants stated that they had unused and expired ARVs at home. Majority of these participants (*n* = 117; 84.2%) indicated that they disposed of the medicines, whilst 12.2% stated that they kept their medicines. A very small percentage of 2.2% stated having used these medicines for decorative purposes whilst 1.4% chose to give their unused and expired ARVs to someone else.

### Participant’s method of disposal (*n* = 117)

**Fig. 1:**
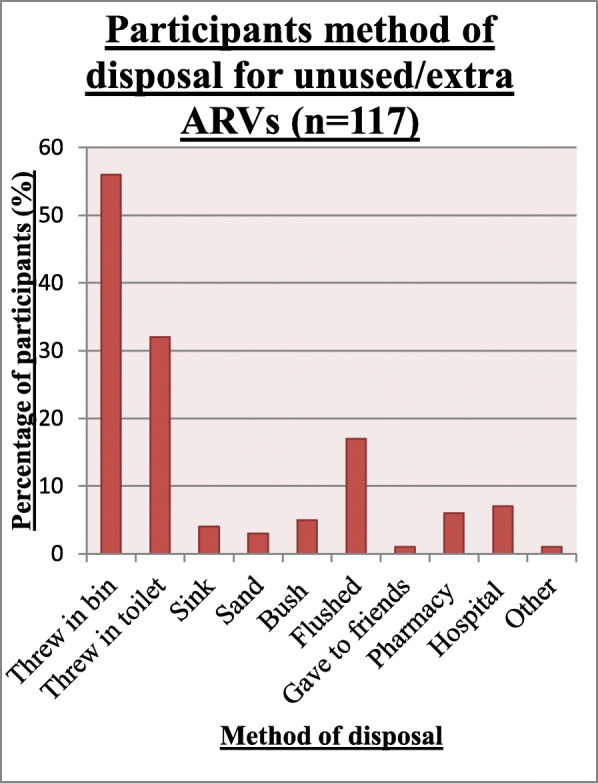
Method of disposal of unused/extra ARVs by HIV infected patients

Of the participants that indicated that they disposed of their unused/expired medicines (*n* = 117; 84.2%), the most common method of disposal was by throwing their unused ARVs into the bin (56.4%) with only 6 and 7% of participants returning their unused ARVs to a hospital or pharmacy respectively (Fig. 1).

Expired ARVs were disposed of similarly with 58 (41.7%) out of 139 participants throwing their expired ARVs in the bin, 23% of participants throwing them in the toilet and only 4.3 and 7.9% of participants returning their expired ARVs to the pharmacy or hospital respectively.

In addition to this, HIV patients were asked if they had other unused and expired medicines at home, 212 (43.8%) participants indicated yes to this question. More than half of these participants (52.4%) had unused and expired pain medicines, 40.1% had flu medicines, 37.3% had antibiotics, 11.3% had anti-hypertensive, 11.3% had Anti-Tuberculosis medicines, 7.1% had anti-diabetic medicines and 4.1% had antidepressants.

**Fig. 2:**
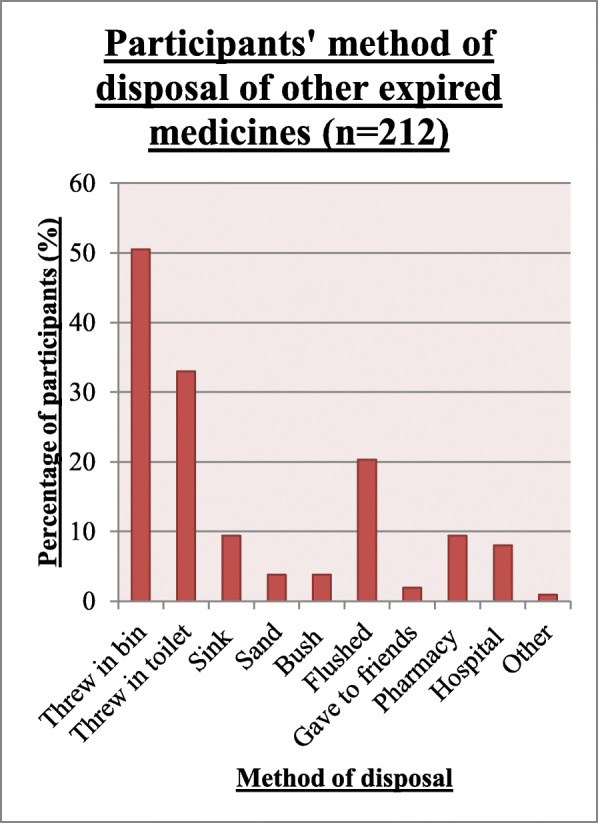
Method of disposal of other expired medicines by HIV infected patients

Just over half of the participants (50.5%) disposed of their expired medicines by throwing them into the bin, 33.0% of participants threw these expired medicines in the toilet, whilst 8 and 9.4% returned their expired medicines to a hospital or pharmacy respectively (Fig. 2). Disposal of unused medicines followed the same pattern.

### Factors influencing participants medicine disposal practices

Participants medicine disposal practices were influenced by their knowledge, with those having appropriate knowledge likely to have proper medicine disposal practices (*p* = 0.002) and those participants who were informed by a doctor had a higher likelihood of safe disposal practices (*p* = 0.020). Participants taking more tablets were to a small degree likely to dispose of their medicines in the sand (*p* = 0.058).

## Discussion

Every human has the constitutional right to an environment that is not harmful to their health and well being [22], environmental health is a growing global concern which requires the identification of hazardous substances and limiting exposure to these substances which could adversely affect human health [23]. Interestingly, in this study 87.8% of participants knew that improper disposal of medicines are harmful to the environment, this was similar to a study done in Serbia where 66.3% of participants agreed that throwing medicines in the bin has detrimental effects on the environment [24]. However, participants understanding of the detrimental effects on the environment did not translate to effective medicine disposal practices, since less than 10% of participants appropriately disposed of their medicines by returning their unused and expired medicines to a hospital or pharmacy. This finding was similar to a study done in Indonesia where only 3% of respondents returned their medicines to a pharmacy [25]. Majority of the participants disposed of their medicine by throwing them into the bin, throwing their medicines and flushing them in the toilet. This result was consistent to the study done in Kabul, where 53.2% of participants disposed of their medicines in the bin and 23.9% flushed their medicines in the toilet [3]. These findings were similar to studies done in countries such as Kuwait, Saudi Arabia, India and the United Kingdom [10, 15, 26, 27].

The study identified that 28.7% of participants had unused and expired ARVs at home and 43.8% of participants had other unused and expired medicines, such as antibiotics, flu, pain and anti -Tuberculosis medicines this finding was similar to a study done in the United states where 56% of participants had unused and expired medicines at home [9]. Of concern was that a small proportion of participants who had unused and expired ARVs at home, chose to keep them at home, this having potential human health risks [27]. However, of interest was the finding that those participants who kept their medicines had proper knowledge on medicine disposal (*p* = 0.041). The possible reason for keeping the medicines could be the distance from their home to either a pharmacy/hospital where they could return it. Some studies state that poor patient compliance, together with over prescribing by doctors and over dispensing by pharmacists are some of the reasons for the accumulation of unwanted and unused medicines in households and eventually in the environment [18]. Some participants were also taking chronic medicines for their diabetic and hypertensive medical condition, increasing the number of tablets they had to take per day. However the number of tablets taken per day did not have a significant correlation with their disposal method except when participants threw the medicines in the sand (*p* < 0.058) there was a slight clinical significance. No other studies could be assessed to confirm or refute this finding.

Of interest was that only 28.3% of participants were aware that South Africa has laws governing the way medicines should be disposed of, similar findings were evident in a study done in Eastern Ethiopia, where 66.9% of the participants did not know about the drug take back system available to them [21]. In South Africa the laws that prescribe the way medicines should be disposed are written in both English and Afrikaans, Afrikaans was not a common language seen among the participants, instead the majority of the participants spoke and understood isiZulu. Hence, participants inability to understand the laws due to the language it is prescribed in could be the reason why majority of the participants were unaware of the laws governing medicine disposal. Furthermore, participants that had secondary or tertiary level of education (*p* = 0.043) with an ability to speak English (*p* = 0.001) were found to be more knowledgeable about the safe disposal of medicines, emphasizing the importance of language as a tool for effective communication and education.

Overall, 62.8% of the participants still lacked knowledge about the proper way to dispose of unused and expired medicines. This indicates that poor patient education and lack of awareness is ultimately resulting in poor disposal methods which is similar to studies done in United states which found that there is a need for patient education [9, 11]. In addition a correlation done between participants’ knowledge and medicine disposal practices found a significant relation between improper medicine disposal practices and poor knowledge and lack of awareness (*p* = 0.002).

Of concern however, was that three quarters of participants in this study were never informed by a healthcare professional about the safe way to dispose of their medicines. This finding is consistent with the Saudi Arabian study where more than 80% of participants were not informed by their healthcare professional on appropriate medicine disposal practices [15]. However, of those participants that were informed by health care professionals were mostly informed by nurses, which could be due to the fact that most ARV clinics in South Africa are currently run by nurses, due to the Nurse Initiative Management of Antiretroviral Therapy (NIMART) programme [28]. In addition to this, this study identified that 71% of participants did not have unused and expired ARVs at home and which could imply that nurses who are in charge of these ARV clinics are ensuring that patients are being counselled effectively to ensure adherence to their ARV treatment regimen. Another interesting finding was that if a doctor informed the participant about medicine disposal, there was a higher likelihood of safe disposal practices by the participant (*p* = 0.020). This could be due to the hierarchy that is prevalent in the health care system where a doctor is always revered for his knowledge when compared to other health care professionals [29]. Irrespective of what designation one has as a health care professional it is absolutely important for them to incorporate methods into their healthcare practice by which they can educate the general patient population to try and limit the presence of these pharmaceuticals in drinking water.

Therefore, patients need to be made more aware about effective medicine disposal methods and the implications of poor disposal practices, in order to encourage them to dispose of their medicines appropriately. In the United States the Food and Drug Administration (FDA) promotes disposal of unused and expired medicines primarily through drug take back programmes, where medicines are returned to pharmacies, however, they also have a list of medicines that can be flushed down the toilet and further recommend that if patients cannot return their medicines then they should mix their medicines with unappealing substance such as cat litter and dispose of it in the bin [30]. South Africa has strict policies with regards to medicine disposal [12], which should be made available to the public in the common spoken languages so that patients become more aware of the laws that govern medicine disposal. Therefore, all stakeholders directly involved with health and environmental issues need to look at current relevant policies and identify how these policies can be amended to ensure proper medicine disposal is being practiced by patients in ways that is convenient and easy for patients to understand and abide to. A similar suggestion to this was made in a Ghanaian study [31]. There is an increased responsibility placed on healthcare professionals, patients, caregivers and other stakeholders to ensure that the medicines that are intended to treat human diseases are not placing any risks onto the environment or human health and should be safely disposed off.

### Limitations

Due to the limited number of study sites used and the sample size, the results of this study cannot be generalised to all HIV infected patients in the province of KwaZulu Natal and South Africa. In addition, this being a self-reported study, the reliability of the information collected cannot be substantiated and the direction of correlation may not be causal in this cross- sectional study.

## Conclusion

This study identified that HIV infected patients and /or their caregivers engage in poor medicine disposal practices and lack adequate knowledge on the safe medicine disposal practices. Thus, there is a need for patient education and public awareness on the proper methods of medicine disposal. This places the responsibility on healthcare professionals together with other stakeholders to ensure that patients are provided with accurate information about disposal of their unused and expired medicines. Proper knowledge will limit the quantity of pharmaceuticals that are entering the environment and causing potential risks to human health.

## Supplementary information

**Additional file 1.**

## Data Availability

The data analysed and used in this study is available on request from the corresponding author.

## Abbreviations

ART: Antiretroviral therapy
ARV: Antiretroviral
FDA: Food and Drug Administration
HIV: Human Immunodeficiency Virus
SPSS: Statistical Package for Social Sciences
WHO: World Health Organisation
NIMART: Nurse Initiative Management of Antiretroviral Therapy

## References

[1] HIV and AIDS in South Africa. AVERT. 2019 [Cited 12 Feb 2019]. Available from: https://www.avert.org/professionals/hiv-around-world/sub-saharan-africa/south-africa.

[2] Ubomba-Jaswa D. Successful HIV treatment create health problems by contaminating water with ARVs. The M&G Online 2019. [Cited 22 Jan 2019]. Available from: https://mg.co.za/article/2018-11-30-00-successful-hiv-treatment-create-health-problems-by-contaminating-water-with-arvs.

[3] Bashaar M, Thawani V, Hassali M, Saleem F (2017). Disposal practices of unused and expired pharmaceuticals among general public in Kabul.

[4] Boxall A (2004). The environmental side effects of medication. EMBO Rep.

[5] Elfaki MG (2014). Immuno-suppression induced by HIV infection. Biol Med.

[6] Paut Kusturica M, Tomas A, Sabo A (2016). Disposal of unused drugs: knowledge and behaviour among people around the world. Rev Environ Contam Toxicol.

[7] Swanepoel C, Bouwman H, Pieters R, Bezuidenhout C (2015). Presence, concentrations and potential implications of HIV-anti-retrovirals in selected water resources in South Africa.

[8] Kelly F, McMillan S, Spinks J, Bettington E, Wheeler A (2018). ‘You don’t throw these things out:’ an exploration of medicines retention and disposal practices in Australian homes. BMC Public Health.

[9] Seehusen D, Edwards J (2006). Patient practices and beliefs concerning disposal of medications. J Am Board Fam Med.

[10] Abahussain E, Ball D, Matowe W (2006). Practice and opinion towards disposal of unused medication in Kuwait. Med Princ Pract.

[11] Kozak M, Melton J, Gernant S, Snyder M (2016). A needs assessment of unused and expired medication disposal practices: a study from the medication safety research network of Indiana. Res Soc Adm Pharm.

[12] South African Government. Medicines and related Substances Control Act, No. 101 of 1965. [cited 09 May 2020]. Available from: http://www.rrfa.co.za/wp-content/uploads/2012/11/Regulations-to-Act-101-published-2003.pdf.

[13] The South African Pharmacy Council (2010). Good pharmacy practice in South Africa. Arcadia.

[14] Health KZN (2019). KZN policy for the disposal of pharmaceutical waste.

[15] AlAzmi A, AlHamdan H, Abualezz R, Bahadig F, Abonofal N, Osman M (2017). Patients’ knowledge and attitude toward the disposal of medications. Aust J Pharm.

[16] Amod F, Chetty K, Essa A, Hlela L, Maharaj C, Oosthuizen F (2019). A pilot study to determine public trends in storage and disposal of medicines: PSSA perspectives journals.

[17] Angi’enda S, Bukachi S (2016). Household knowledge and perceptions on disposal practices of unused medicines in Kenya. J Anthropol Archaeol.

[18] Mashiane M (2017). Disposal practices for unwanted Medicinesfrom households in Johannesburg.

[19] Shukla T, Bajaj R, Khanna S, Pandey S, Dubey R, Upmanyu N (2017). Role of pharmacist in pharmaceutical waste management. World J Environ Biosci.

[20] KwaZulu-Natal Municipalities. 2019 [cited 1 October 2019]. Available from: https://municipalities.co.za/provinces/view/4/kwazulu-natal.

[21] Ayele Y, Mamu M (2018). Assessment of knowledge, attitude and practice towards disposal of unused and expired pharmaceuticals among community in Harar city, eastern Ethiopia. J Pharm Policy Pract.

[22] South African Government (1996). Constitution of the Republic of South Africa - chapter 2: bill of rights.

[23] National Environmental Health Association: NEHA (2019). Definitions of environmental Health.

[24] Paut Kusturica M, Tomas A, Tomic Z, Bukumiric D, Corac A, Horvat O (2016). Analysis of expired medications in Serbian households. Slovenian J Public Health.

[25] Kristina S, Wiedyaningsih C, Cahyadi A, Ridwan B (2018). A survey on medicine disposal practice among households in Yogyakarta. Asian J Pharm.

[26] N S, Jha A. Knowledge and awareness regarding safe drug disposal system among general population of India. Aust J Pharm. 2018;06(02): 1–4.

[27] Bound J, Voulvoulis N (2005). Household disposal of pharmaceuticals as a pathway for aquatic contamination in the United Kingdom. Environ Health Perspect.

[28] KZN Health (2019). Nurse initiated Management of Antiretroviral therapy (NIMART) – ‘the KZN success story’.

[29] Eaton R (2019). Hierarchy in the medical field.

[30] United States Food and Drug administration (2019). How to dispose of unused medicines.

[31] Udofia E, Gulis G, Fobil J (2017). Solid medical waste: a cross sectional study of household disposal practices and reported harm in southern Ghana. BMC Public Health.

