# Supplementary material for: “Knowledge and practices of HIV infected patients regarding medicine disposal among patients attending public ARV clinics in KwaZulu Natal, South Africa”
Source: BMC Public Health. 2020 Jun 8;20:884. doi: 10.1186/s12889-020-09018-4 (PMC7282097; doi:10.1186/s12889-020-09018-4)
Supplement: Supplementary file 1 — Additional file 1. [file 12889_2020_9018_MOESM1_ESM.pdf]

APPENDIX 1

QUESTIONNAIRE

Dear Participant

Thank you for taking your time to complete this questionnaire.

Please inform the researchers about your language of choice (English/Zulu) so that the questionnaire, in your preferred language, could be administered.

Please fill the questionnaire by making a cross (X) or a tick (✓) in the appropriate box. Please fill in the form as honestly and accurately as possible.

---

1. **GENDER**

MALE	FEMALE	TRANSGENDER

2. **AGE**

18-20	21-30	31-40	41-50	51-60	>60

3. **EDUCATIONAL LEVEL**

NO FORMAL SCHOOLING	PRIMARY	SECONDARY	TERTIARY

4. **LANGUAGE YOU SPEAK**(Please check the appropriate box-more than 1 box can be checked.)

**English** ☐	**Tswana** ☐	**Ndebele** ☐
**Zulu** ☐	**Swati** ☐	**Xhosa** ☐
**Tsonga** ☐	**Southern Sotho** ☐	**Venda** ☐
**Afrikaans** ☐	**Northern Sotho** ☐	**Other** ☐

If other, please specify: ____

5. What type of area do you live in?

**URBAN**	**SEMI URBAN**	**SEMI RURAL**	**RURAL**

6. Where do you live [your accommodation]

**FORMAL HOUSE**	**INFORMAL HOUSE**	**APARTMENT/FLAT**	**OTHER** (Please specify)

7. Do you have easy access to water?

YES ☐ **NO** ☐

8. If yes, is water available inside your home?

YES ☐ **NO** ☐

9. If no, how do you obtain water?

**RIVER**	**BOREHOLE**	**MUNICIPAL TAP**	**TANKER**	**OTHER** (Please specify)

10. What sewage system do you have at home?

**PIT TOILETS**	**FLUSH TOILETS**	**COMMUNAL --OTHER** (Please specify)

11. How far is the nearest pharmacy/clinic/hospital from your home in kilometres?

**<2or it is near**	**2-4**	**5-10**	**11-15**	**>15**	**Don't know**

12. How many tablets do you take a day? HIV and other medicines

____

13. Have you ever had unused/extra or expired HIV medicines at home?

YES ☐ **NO** ☐

14. If YES what did/do you do with it?

Keep it/kept it ☐
Give/gave it to someone ☐
Use it as decorative ☐
Disposed of it/ throw/threw it ☐

IF YOU DISPOSED OR THREW IT :

15. How did you do this

Threw them in the bin	YES ☐	NO ☐
Threw them in the toilet	YES ☐	NO ☐
Rinsed them down the sink	YES ☐	NO ☐
Buried them in the sand	YES ☐	NO ☐
Threw them in the bush/field	YES ☐	NO ☐
Flushed them down the toilet	YES ☐	NO ☐
Gave them to friends and family	YES ☐	NO ☐
Returned them to a pharmacy	YES ☐	NO ☐
Returnedit to a hospital	YES ☐	NO ☐

Other ☐(If other, please state)

____

16. How have you been disposing of your expired ARVs?

Throwing them in the bin	YES ☐	NO ☐
Throwing them in the toilet	YES ☐	NO ☐
Rinsing them down the sink	YES ☐	NO ☐
Burying them in sand	YES ☐	NO ☐
Throwing them in the bush/field	YES ☐	NO ☐
Flushing them down the toilet	YES ☐	NO ☐
Giving them to friends and family	YES ☐	NO ☐
Returning them to a pharmacy	YES ☐	NO ☐
Returning it to a hospital	YES ☐	NO ☐

Other ☐(If other, please state)

---

17. Have you ever had other unused or expired medicines at home?

YES ☐ **NO** ☐

---

18. What kind of medicines were these?

ANTIBIOTICS ☐
(Amoxicillin, Ciprofloxacin, Bactrim)

ANTI-DEPRESSANTS ☐
(Escitalopram, Fluoxetine, Sertaline)

PAIN ☐
(Ibuprofen, panado)

FLU ☐

ANTI-DIABETIC ☐
(Metformin)

ANTI-HPYERTENSIVE ☐
(Hydrochlorothiazide, pharmapress)

TB ☐

OTHER ☐
(If other, please state the medication)

19. How have you been disposing of the above unused/extra medicines?

Throwing them in the bin	YES ☐	NO ☐
Throwing them in the toilet	YES ☐	NO ☐
Rinsing them down the sink	YES ☐	NO ☐
Burying them in sand	YES ☐	NO ☐
Throwing them in the bush/field	YES ☐	NO ☐
Flushing them down the toilet	YES ☐	NO ☐
Giving them to friends and family	YES ☐	NO ☐
Returning them to a pharmacy	YES ☐	NO ☐
Returning it to a hospital	YES ☐	NO ☐

Other ☐(If other, please state)

---

20. How have you been disposing of the above stated expired medicines?

Throwing them in the bin	YES ☐	NO ☐
Throwing them in the toilet	YES ☐	NO ☐
Rinsing them down the sink	YES ☐	NO ☐
Burying them in sand	YES ☐	NO ☐
Throwing them in the bush/field	YES ☐	NO ☐
Flushing them down the toilet	YES ☐	NO ☐
Giving them to friends and family	YES ☐	NO ☐
Returning them to a pharmacy	YES ☐	NO ☐
Returning it to a hospital	YES ☐	NO ☐

Other ☐(If other, please state)

---

21. Have you ever been informed about how to dispose of your medicines?

YES ☐ **NO** ☐

---

22. Who informed you?

NURSE ☐

DOCTOR ☐

PHARMACIST ☐

OTHER ☐

If other, please specify

---

23. What do you think is the best way to dispose of unused or expired medicines?

Throwing them in the bin	YES ☐	NO ☐
Throwing them in the toilet	YES ☐	NO ☐
Rinsing them down the sink	YES ☐	NO ☐
Burying them in sand	YES ☐	NO ☐
Throwing them in the bush/field	YES ☐	NO ☐
Flushing them down the toilet	YES ☐	NO ☐
Giving them to friends and family	YES ☐	NO ☐
Returning them to a pharmacy	YES ☐	NO ☐
Returning to a hospital	YES ☐	NO ☐
Other	YES ☐	NO ☐

(If other, please specify)

---

24. Do you think medicines are harmful to the environment if disposed of incorrectly?

YES ☐ **NO** ☐

---

25. Do you know if there is a law that informs you on how to dispose of medicines?

YES ☐ **NO** ☐
